# Supplementary material for: iSoMAs: Finding isoform expression and somatic mutation associations in human cancers
Source: PLoS Comput Biol. 2025 Mar 7;21(3):e1012847. doi: 10.1371/journal.pcbi.1012847 (PMC12052144; doi:10.1371/journal.pcbi.1012847)
Supplement: S1 Fig — (A) Cluster residence heatmap shows the number of samples from a given Isoform cluster (BRCA Cluster) that overlaps with each of the five classic breast cancer subtypes (BRCA Subtype) including Basal, HER2E (HER2-enriched), LumA, LumB and normal-like based on PAM50 mRNA profiles (Thennavan et al, 2021) [21]. This overlap analysis includes 1,198 out of 1,215 TCGA-BRCA samples with subtype information available. True Normal: adjacent normal tissue samples; CLOW: Claudin-low samples. (B) Cluster residence heatmap shows the number of samples from a given cancer type that reside within each of the 22 (0–21) annotated Mutation clusters. (C) Somatic mutation clusters shown on the Isoform UMAP map. Numbers denote indexes of the Mutation clusters. (D) Tumor samples are shown on the Isoform UMAP map and are colored according to clustering results (Hoadley et al. 2018) [22] based on various TCGA data types as indicated. Numbers denote indexes of the clusters in each clustering scheme. (E) Tumor samples are shown on the Isoform UMAP map and are colored by tumor stage, tumor purity (Aran et al. 2015) [45] and cancer type. In (C-E), NA refers to samples absent from those used to generate the Isoform UMAP (Fig 1C). (DOCX) [file pcbi.1012847.s001.docx]

**S1 Fig. TCGA multi-omics data.** Related to Figure 1.

(A) Cluster residence heatmap shows the number of samples from a given Isoform cluster (BRCA Cluster) that overlaps with each of the five classic breast cancer subtypes (BRCA Subtype) including Basal, HER2E (HER2-enriched), LumA, LumB and normal-like based on PAM50 mRNA profiles (Thennavan et al, 2021). This overlap analysis includes 1,198 out of 1,215 TCGA-BRCA samples with subtype information available. True Normal: adjacent normal tissue samples; CLOW: Claudin-low samples.

(B) Cluster residence heatmap shows the number of samples from a given cancer type that reside within each of the 22 (0–21) annotated Mutation clusters.

(C) Somatic mutation clusters shown on the Isoform UMAP map. Numbers denote indexes of the Mutation clusters.

(D) Tumor samples are shown on the Isoform UMAP map and are colored according to clustering results (Hoadley et al. 2018) based on various TCGA data types as indicated. Numbers denote indexes of the clusters in each clustering scheme.

(E) Tumor samples are shown on the Isoform UMAP map and are colored by tumor stage, tumor purity (Aran et al. 2015) and cancer type.

In (C-E), NA refers to samples absent from those used to generate the Isoform UMAP (Figure 1C).
