# Supplementary material for: iSoMAs: Finding isoform expression and somatic mutation associations in human cancers
Source: PLoS Comput Biol. 2025 Mar 7;21(3):e1012847. doi: 10.1371/journal.pcbi.1012847 (PMC12052144; doi:10.1371/journal.pcbi.1012847)
Supplement: S2 Fig — (A) Bar plot shows the number of genes tested significant along each of the 50 PC-axes in each cancer type. A gene can be counted multiple times if it tested significant along multiple PC axes. The lower panel is a zoom-in of the upper panel and plots the last 10 PCs (PC_41 – PC_50) as indicated. (B) Scaled expression levels of the top 50 positive and top 50 negative isoforms of all samples ordered by PC scores along PC_2 for TP53 in LUAD cancer. The isoforms were ranked by the PC loading value along PC_2 axis. (C) Approximation of the PC score along PC_2 with the top isoforms as indicated by the x-labels in LUAD. The total number of variable isoforms for LUAD cancer is 3,315, as shown in the rightmost panel. (D) Direct differential isoform expression analysis on the top 50 positive (upper) and top 50 negative (lower) isoforms (ranked by PC loading) based on TP53 mutation status. (E) Direct differential isoform expression analysis on all isoforms of the top four associated genes in (B), including positively associated TPX2 and CCNB1, and negatively associated SELENBP1 and C16orf89, based on TP53 mutation status. (F) The details of differential PC score analysis along all 50 PC axes for TP53 in LUAD cancer. TP53 was tested significant along four PC axes: PC_2, PC_4, PC_5 and PC_50 in LUAD. (G) Bar plot shows the proportion of variance explained by each of the first 100 PCs in LUAD cancer. (H) Similar to (B), heatmaps show the scaled expression levels of the top 50 positive and top 50 negative isoforms of all samples ordered by PC scores along each PC axis (PC_4, PC_5 or PC_50 as indicated) for TP53 in LUAD cancer. The isoforms were ranked by the PC loading values along each corresponding PC axis. (I) Left: Spearman correlation between PC score (along PC_4, PC_5 or PC_50 axis as indicated) and expression level of all input 3,315 isoforms in LUAD cancer. Right: Association between TP53 mutation and expression level of all input 3,315 isoforms in LUAD cancer, measured b [file pcbi.1012847.s002.docx]

**S2 Fig. Overview and close-up look of the iSoMAs genes.** Related to Figure 2.

(A) Bar plot shows the number of genes tested significant along each of the 50 PC-axes in each cancer type. A gene can be counted multiple times if it tested significant along multiple PC axes. The lower panel is a zoom-in of the upper panel and plots the last 10 PCs (PC_41 – PC_50) as indicated.

(B) Scaled expression levels of the top 50 positive and top 50 negative isoforms of all samples ordered by PC scores along PC_2 for TP53 in LUAD cancer. The isoforms were ranked by the PC loading value along PC_2 axis.

(C) Approximation of the PC score along PC_2 with the top isoforms as indicated by the x-labels in LUAD. The total number of variable isoforms for LUAD cancer is 3,315, as shown in the rightmost panel.

(D) Direct differential isoform expression analysis on the top 50 positive (upper) and top 50 negative (lower) isoforms (ranked by PC loading) based on TP53 mutation status.

(E) Direct differential isoform expression analysis on all isoforms of the top four associated genes in (B), including positively associated TPX2 and CCNB1, and negatively associated SELENBP1 and C16orf89, based on TP53 mutation status.

(F) The details of differential PC score analysis along all 50 PC axes for TP53 in LUAD cancer. TP53 was tested significant along four PC axes: PC_2, PC_4, PC_5 and PC_50 in LUAD.

(G) Bar plot shows the proportion of variance explained by each of the first 100 PCs in LUAD cancer.

(H) Similar to (B), heatmaps show the scaled expression levels of the top 50 positive and top 50 negative isoforms of all samples ordered by PC scores along each PC axis (PC_4, PC_5 or PC_50 as indicated) for TP53 in LUAD cancer. The isoforms were ranked by the PC loading values along each corresponding PC axis.

(I) Left: Spearman correlation between PC score (along PC_4, PC_5 or PC_50 axis as indicated) and expression level of all input 3,315 isoforms in LUAD cancer. Right: Association between TP53 mutation and expression level of all input 3,315 isoforms in LUAD cancer, measured by Wilcoxon rank-sum test. The top 50 positive and top 50 negative isoforms (ranked by PC loadings along each PC axis) are marked red and green, respectively.

(J) Boxplot shows the number of iSoMAs target genes with dual-direction association with mutation status of each iSoMAs gene detected in each cancer type. The iSoMAs gene with largest number of dual-direction associated genes is labeled for each cancer type.

(K) Boxplot shows the Log10-transformed average HMP (Log10mHMP) for each iSoMAs gene in each cancer type. The iSoMAs gene with highest Log10mHMP value is labeled for each cancer type.

(L) Specific examples showing dual-direction associations for representative iSoMAs genes as labeled in (G). For each labeled iSoMAs gene, the dual-direction associated gene with the largest Log10mHMP value is chosen to show. For each dual-direction associated gene, only isoforms with Wilcoxon rank-sum test P<0.05 are shown for simplicity.

In (J-K), the x-axis labels the TCGA cancer types and the number of iSoMAs genes with at least one dual-direction association within their top 100 target isoforms in each cancer type.

In (D-F) and (L), significance levels were derived from Wilcoxon rank-sum test, *****P*<1e-4, ****P*<1e-3, ***P*<0.01, **P*<0.05, ns: not significant.
