# Supplementary material for: iSoMAs: Finding isoform expression and somatic mutation associations in human cancers
Source: PLoS Comput Biol. 2025 Mar 7;21(3):e1012847. doi: 10.1371/journal.pcbi.1012847 (PMC12052144; doi:10.1371/journal.pcbi.1012847)
Supplement: S3 Fig — (A) Detailed chromosome distribution and chromosome preference profiles of top 100 target isoforms of representative iSoMAs genes TP53 (left) and KRAS (right) across cancer types. P-values were derived from KS test for chromosome preference and are indicated above corresponding cancer types. Lhg19: the chromosome length distribution of the 24 chromosomes; Niso: number distribution of all isoforms across the 24 chromosomes. (B) Schematic of the hypergeometric test performed to determine cis-regulation of iSoMAs genes. (C) Number of cis-regulation iSoMAs genes determined at various significance levels for each cancer type as indicated. The cancer types, together with the number of iSoMAs genes with P>0.999 (potentially trans-regulation iSoMAs genes) and the number of all iSoMAs genes detected in each cancer type are indicated in the x-axis. (DOCX) [file pcbi.1012847.s003.docx]

**S3 Fig. More details on cis- *vs.* trans-regulation of iSoMAs genes.** Related to Figure 3.

(A) Detailed chromosome distribution and chromosome preference profiles of top 100 target isoforms of representative iSoMAs genes TP53 (left) and KRAS (right) across cancer types. P-values were derived from KS test for chromosome preference and are indicated above corresponding cancer types. Lhg19: the chromosome length distribution of the 24 chromosomes; Niso: number distribution of all isoforms across the 24 chromosomes.

(B) Schematic of the hypergeometric test performed to determine cis-regulation of iSoMAs genes.

(C) Number of cis-regulation iSoMAs genes determined at various significance levels for each cancer type as indicated. The cancer types, together with the number of iSoMAs genes with *P*>0.999 (potentially trans-regulation iSoMAs genes) and the number of all iSoMAs genes detected in each cancer type are indicated in the x-axis.
