# Supplementary material for: iSoMAs: Finding isoform expression and somatic mutation associations in human cancers
Source: PLoS Comput Biol. 2025 Mar 7;21(3):e1012847. doi: 10.1371/journal.pcbi.1012847 (PMC12052144; doi:10.1371/journal.pcbi.1012847)
Supplement: S4 Fig — (A) Signaling pathway enrichment analysis on the top 908 iSoMAs genes that tested significant in ≥3 cancer types against 50 MSigDB Hallmark gene sets in both pan-cancer (bar plot, left) and cancer-specific (heatmap, right) manners. Gene numbers were trimmed to the top 908 (still subject to P<0.05 in the iSoMAs analysis) in the cancer-specific enrichment analyses for a fair comparison. Number of genes in each gene set (n) and number of iSoMAs genes hitting that gene set (m) are shown on each bar (m/n). Cell migration and proliferation related gene sets are marked. (B) Univariate and multivariate Cox regression analysis on the iSoMAs gene mutation and its corresponding PC score across pan-cancer. iSoMAs: number of all iSoMAs genes detected in each cancer type; Test: number of cancer-specific iSoMAs genes overlapping with the 984 pan-cancer iSoMAs genes; Significant: number of iSoMAs genes tested significant in either univariate or multivariate Cox regression analysis in each cancer type; Additive: number of iSoMAs genes for which the multivariate analysis yielded higher significance level (or smaller p-value) compared to the univariate regression analysis. (C) Paired t-test compares the p-values between univariate and multivariate regression analysis for mutation (left) and its corresponding PC score (right) of the Additive iSoMAs genes in BLCA cancer. (D) Survival analysis based on the Additive iSoMAs gene PRKDC in BLCA cancer. BLCA samples were divided into groups based on the mutation status of the gene (left), the sign of its corresponding PC score (middle) and combination of them (right). Mutation: 0=wildtype, 1=mutant; PC: 0=negative, 1=positive PC score. MutPC: Combination Mutation_PC score. Sample size of each group is indicated. P-values were derived from log-rank test. (DOCX) [file pcbi.1012847.s004.docx]

**S4 Fig. More results on biological and clinical significance of the iSoMAs genes.** Related to Figure 4.

(A) Signaling pathway enrichment analysis on the top 908 iSoMAs genes that tested significant in ≥3 cancer types against 50 MSigDB Hallmark gene sets in both pan-cancer (bar plot, left) and cancer-specific (heatmap, right) manners. Gene numbers were trimmed to the top 908 (still subject to *P*<0.05 in the iSoMAs analysis) in the cancer-specific enrichment analyses for a fair comparison. Number of genes in each gene set (n) and number of iSoMAs genes hitting that gene set (m) are shown on each bar (m/n). Cell migration and proliferation related gene sets are marked.

(B) Univariate and multivariate Cox regression analysis on the iSoMAs gene mutation and its corresponding PC score across pan-cancer. iSoMAs: number of all iSoMAs genes detected in each cancer type; Test: number of cancer-specific iSoMAs genes overlapping with the 984 pan-cancer iSoMAs genes; Significant: number of iSoMAs genes tested significant in either univariate or multivariate Cox regression analysis in each cancer type; Additive: number of iSoMAs genes for which the multivariate analysis yielded higher significance level (or smaller p-value) compared to the univariate regression analysis.

(C) Paired t-test compares the p-values between univariate and multivariate regression analysis for mutation (left) and its corresponding PC score (right) of the Additive iSoMAs genes in BLCA cancer.

(D) Survival analysis based on the Additive iSoMAs gene PRKDC in BLCA cancer. BLCA samples were divided into groups based on the mutation status of the gene (left), the sign of its corresponding PC score (middle) and combination of them (right). Mutation: 0=wildtype, 1=mutant; PC: 0=negative, 1=positive PC score. MutPC: Combination Mutation_PC score. Sample size of each group is indicated. P-values were derived from log-rank test.
