# Supplementary material for: iSoMAs: Finding isoform expression and somatic mutation associations in human cancers
Source: PLoS Comput Biol. 2025 Mar 7;21(3):e1012847. doi: 10.1371/journal.pcbi.1012847 (PMC12052144; doi:10.1371/journal.pcbi.1012847)
Supplement: S5 Fig — Shown are the top 20 frequently mutated RBP (A) and TF (B) genes in each of the four cancer types shown in Fig 5D. Bars on the top indicate the number of mutations (Log10-transformed) detected in the top 20 RBP genes in each tumor sample. Bars on the right indicate the minimum p-value (Log10-transformed) along the 50 PCs obtained in the iSoMAs analysis for each gene detected as iSoMAs gene in corresponding cancer type. (DOCX) [file pcbi.1012847.s005.docx]

**S5 Fig. Mutational landscape of the top RBP and TF genes in representative cancer types.** Related to Figure 5.

Shown are the top 20 frequently mutated RBP (A) and TF (B) genes in each of the four cancer types shown in Figure 5D. Bars on the top indicate the number of mutations (Log10-transformed) detected in the top 20 RBP genes in each tumor sample. Bars on the right indicate the minimum p-value (Log10-transformed) along the 50 PCs obtained in the iSoMAs analysis for each gene detected as iSoMAs gene in corresponding cancer type.
