# Supplementary material for: iSoMAs: Finding isoform expression and somatic mutation associations in human cancers
Source: PLoS Comput Biol. 2025 Mar 7;21(3):e1012847. doi: 10.1371/journal.pcbi.1012847 (PMC12052144; doi:10.1371/journal.pcbi.1012847)
Supplement: S6 Fig — (A) Frequency distribution of Log10-HMP values for gene pairs from both iSoMAs (iSoMAs+) and non-iSoMAs (iSoMAs-) pools in 33 cancer types. (B) Frequency distribution of Log10-HMP values for gene pairs from iSoMAs+ pool only in 33 cancer types. (C) Frequency distribution of Log10-HMP values for gene pairs from iSoMAs- pool only in 33 cancer types. The number of gene pairs for each scenario is shown above each panel. The p-values were derived from Kolmogorov-Smirnov (KS) test. (DOCX) [file pcbi.1012847.s006.docx]

**S6 Fig. More details on co-profiles of chromatin accessibility surrounding iSoMAs gene-target pairs.** Related to Figure 6.

(A) Frequency distribution of Log10-HMP values for gene pairs from both iSoMAs (iSoMAs+) and non-iSoMAs (iSoMAs-) pools in 33 cancer types.

(B) Frequency distribution of Log10-HMP values for gene pairs from iSoMAs+ pool only in 33 cancer types.

(C) Frequency distribution of Log10-HMP values for gene pairs from iSoMAs- pool only in 33 cancer types.

The number of gene pairs for each scenario is shown above each panel. The p-values were derived from Kolmogorov-Smirnov (KS) test.
