# Supplementary material for: iSoMAs: Finding isoform expression and somatic mutation associations in human cancers
Source: PLoS Comput Biol. 2025 Mar 7;21(3):e1012847. doi: 10.1371/journal.pcbi.1012847 (PMC12052144; doi:10.1371/journal.pcbi.1012847)
Supplement: S7 Fig — (A) Differential isoform expression analysis on four genes as indicated, based on SF3B1 mutation status in UVM cancer. (B) Differential isoform expression analysis on ABCC5 and UQCC based on SF3B1 mutation status in UCEC cancer. (C) Survival analysis based on the mutation status of SF3B1 in UCEC cancer. (D) Differential isoform expression analysis on NUMB based on RBM10 mutation status in STAD cancer. (E) Survival analysis based on the mutation status of RBM10 in STAD cancer. (F) Differential isoform expression analysis on NUMB based on RBM10 mutation status in UCEC cancer. (G) Survival analysis based on the mutation status of RBM10 in UCEC cancer. P-values for survival analysis were derived from log-rank test; Significance levels for differential analysis were derived from Wilcoxon rank-sum test, ****P<1e-4, ***P<1e-3, **P<0.01, *P<0.05, ns: non-significant. (DOCX) [file pcbi.1012847.s007.docx]

**S7 Fig. Additional validation results for iSoMAs genes SF3B1, RBM10 and TP53.** Related to Figure 7.

(A) Differential isoform expression analysis on four genes as indicated, based on SF3B1 mutation status in UVM cancer.

(B) Differential isoform expression analysis on ABCC5 and UQCC based on SF3B1 mutation status in UCEC cancer.

(C) Survival analysis based on the mutation status of SF3B1 in UCEC cancer.

(D) Differential isoform expression analysis on NUMB based on RBM10 mutation status in STAD cancer.

(E) Survival analysis based on the mutation status of RBM10 in STAD cancer.

(F) Differential isoform expression analysis on NUMB based on RBM10 mutation status in UCEC cancer.

(G) Survival analysis based on the mutation status of RBM10 in UCEC cancer.

P-values for survival analysis were derived from log-rank test; Significance levels for differential analysis were derived from Wilcoxon rank-sum test, *****P*<1e-4, ****P*<1e-3, ***P*<0.01, **P*<0.05, ns: non-significant.
