# Supplementary material for: iSoMAs: Finding isoform expression and somatic mutation associations in human cancers
Source: PLoS Comput Biol. 2025 Mar 7;21(3):e1012847. doi: 10.1371/journal.pcbi.1012847 (PMC12052144; doi:10.1371/journal.pcbi.1012847)
Supplement: S8 Fig — (A) Differential expression analysis on NCI-H1975 lung cancer cell line data for top (upper panel) and bottom (middle panel) 25 isoform targets of TP53 ranked by feature loadings derived from iSoMAs analysis on TCGA-LUAD, as well as randomly chosen 25 isoforms (lower panel) from outside the iSoMAs input. TP53 Mutant group corresponds to the original H1975 lung cancer cell line samples (n=4), TP53 WildType group refers to the samples treated with SCH529074 compound (n=4). (B) Gene structure (hg19) of specific isoforms of TPX2 and NCAPG in the TCGA isoform expression data. (C) Survival analysis based on the expression level of representative positive (left panel) and negative (right panel) isoform targets of TP53 in LUAD. LUAD tumor samples were divided into 3 tiers equally based on expression level of each isoform, and only the high (n=171) and low (n=171) tiers were kept for comparison. (D) Differential expression analysis for representative positive (left panel) and negative (right panel) isoform targets of TP53 between normal (n=59) and tumor (n=517) LUAD samples. (E) Cell cycle analysis results for NCI-H1975 cells under various treatment conditions at different time points as indicated. (F) Cell apoptosis analysis for NCI-H1975 cells under various treatment conditions at different time points as indicated. P-values for survival analysis were derived from log-rank test; Significance levels for differential analysis were derived from Wilcoxon rank-sum test (for TCGA data) or t-test (for H1975 cell line data), ****P<1e-4, ***P<1e-3, **P<0.01, *P<0.05, ns: non-significant. (DOCX) [file pcbi.1012847.s008.docx]

**S8 Fig. Additional details for wet-lab experiments in NCI-H1975 lung cancer cell line.** Related to Figure 8.

(A) Differential expression analysis on NCI-H1975 lung cancer cell line data for top (upper panel) and bottom (middle panel) 25 isoform targets of TP53 ranked by feature loadings derived from iSoMAs analysis on TCGA-LUAD, as well as randomly chosen 25 isoforms (lower panel) from outside the iSoMAs input. TP53 Mutant group corresponds to the original H1975 lung cancer cell line samples (n=4), TP53 WildType group refers to the samples treated with SCH529074 compound (n=4).

(B) Gene structure (hg19) of specific isoforms of TPX2 and NCAPG in the TCGA isoform expression data.

(C) Survival analysis based on the expression level of representative positive (left panel) and negative (right panel) isoform targets of TP53 in LUAD. LUAD tumor samples were divided into 3 tiers equally based on expression level of each isoform, and only the high (n=171) and low (n=171) tiers were kept for comparison.

(D) Differential expression analysis for representative positive (left panel) and negative (right panel) isoform targets of TP53 between normal (n=59) and tumor (n=517) LUAD samples.

(E) Cell cycle analysis results for NCI-H1975 cells under various treatment conditions at different time points as indicated.

(F) Cell apoptosis analysis for NCI-H1975 cells under various treatment conditions at different time points as indicated.

P-values for survival analysis were derived from log-rank test; Significance levels for differential analysis were derived from Wilcoxon rank-sum test (for TCGA data) or *t*-test (for H1975 cell line data), ****P<1e-4, ***P<1e-3, **P<0.01, *P<0.05, ns: non-significant.
