# Supplementary material for: iSoMAs: Finding isoform expression and somatic mutation associations in human cancers
Source: PLoS Comput Biol. 2025 Mar 7;21(3):e1012847. doi: 10.1371/journal.pcbi.1012847 (PMC12052144; doi:10.1371/journal.pcbi.1012847)
Supplement: Attachment — Submitted filename: Response letter.docx [file pcbi.1012847.s018.docx]

**Response to reviewers’ comments**

Reviewer #1: Tan et al. demonstrate iSoMAs, a novel computational pipeline based on principal component analysis (PCA), to investigate the association between gene somatic mutations and isoform expression in TCGA cancer data. The pipeline involved two primary steps:

1. The high-dimensional isoform expression matrix was refined using the mean.var.plot method from Seurat to retain only the most variable isoforms. This high-variable isoform matrix was then further compressed to 50 dimensions via PCA. Each principal component (PC) represented a linear combination of isoforms, referred to as meta-isoforms.

2. A Wilcoxon rank-sum test was performed separately for each of the 50 pcs according to the individual mutation status of each SNV. The Bonferroni correction procedure was then applied at the PC level for multiple testing corrections.

The authors applied iSoMAs to 33 TCGA cancer types and aggregated all 7,140 significant genes into a unique iSoMAs gene set. There are 908 iSoMAs genes identified in three or more cancer types, while 4,582 genes are exclusive to one cancer type. Statistical analyses revealed that iSoMAs target isoforms showed no preference between cis- and trans-acting mechanisms, involving well-known oncogenes, tumor suppressors, RNA-binding proteins, and splicing factors. Additionally, the authors also performed wet-lab experiments to validate the top iSoMAs gene TP53.

The author has proposed an innovative idea and developed an efficient iSoMAs algorithm capable of capturing cross-chromosomal correlations. However, further analysis and explanation are needed regarding the robustness, interpretability, and statistical analysis of the multiple testing correction issue. Thus, our suggestion is "major revision".

Major comments:
1. The authors trimmed an approximately 70,000-dimensional whole-genome isoform expression matrix to 3,667 dimensions using the mean.var.plot method from Seurat and then further reduced the dimensions to 50 using PCA, instead of directly associating SNV grouping of a specific gene with the expression of certain isoforms. The association is between mutated genes and meta-isoforms (linear combinations of isoforms). Several questions need to be answered here:

a. Seurat is designed for single-cell expression data where the read counts could be sparser and noisier, is there a specific reason using it for the TCGA individual level bulk seq data? And the default method for identifying high variable genes in Seurat is vst. Why choose the mean.var.plot method to select highly variable isoforms? How was the number 3,667 determined? Are iSoMAs genes robust for the method of selecting highly variable isoforms?

Reply:

Thank you for raising this important point. We first clarify that while Seurat is designed for single-cell genomics data analysis, the authors of Seurat have stated that it is safe to use Seurat for bulk RNA-seq analysis (<https://github.com/satijalab/seurat/issues/826>).

We further clarify that we only used part of the Seurat functions (i.e., normalization, scaling, clustering, and dimension reduction) whose underlying algorithms are based on general mathematics/principles and for general purposes, not specifically designed for the single-cell data. We chose the Seurat package to perform these basic bioinformatic and statistical analyses because it is a well-established and structuralized toolset which is convenient to operate.

The major reason we chose ‘mean.var.plot’ (mvp) method instead of the default ‘vst’, as we now explicitly explain in the manuscript, is that ‘mvp’ considers more about the relationship between variability and average expression of each isoform across samples compared to ‘vst’. This choice ensures that the selected highly variable genes are not just those with high variance but also have substantial mean expression levels. This balance helps in identifying biologically significant genes and isoforms that contribute substantially to cancer type-specific functions or states. It’s reasonable that different parameters could select different candidate isoforms, and as above explained, the ‘mvp’ method is better for prioritizing those biologically meaningful genes for downstream analysis.

Another reason that we chose the ‘mvp’ method to perform the initial screening is that it gives a specific number of candidates after computing a scaled dispersion value for each feature, with all features of dispersion.scaled<1 automatically discarded. And that’s exactly how the number of **3,667** most variable features (in the joint analysis of all TCGA samples) was determined. In contrast, the ‘vst’ method calculates a standardized variance for each feature without any further recommendation. The users must rank the variances and select a specific number of top features by themselves (via an additional parameter ‘nfeatures’), which is inconvenient and subjective to users.

b. Why was the number of dimensions reduced to 50PCs? What if fewer PCs were selected, such as 10 or 20, or if the dimensions were increased to 100? Beyond the number of iSoMAs genes, we think it is also crucial to assess whether the same iSoMAs genes would consistently appear and how their PC loadings would change. Since the current target isoforms seem to be distributed genome-wide without chromosomal preference, we are curious whether the target isoforms (and their PC loading weights) for the same iSoMAs gene would vary drastically with different parameter settings.

Reply:

Basically, the number of dimensions was reduced to 50 PCs as default in the Seurat package. In PCA, the original input matrix was transformed into a low-dimensional matrix (the PC score matrix) whose dimension is strictly smaller than that of the original one, by calculating a PC loading matrix (Figure R1). Therefore, a complete PCA transformation (decomposition) yields as many PCs as the smaller dimension of the original matrix minus 1. Taking LUAD cancer (blue numbers in Figure R1) as an example, the dimension of 517*3315 of the original matrix was reduced to the PC score matrix of 517*516 in a complete PCA transformation. This transformation is fixed, in the sense that the PC scores along any PC axis exactly correspond to the PC loadings along that same PC axis, without affecting values along any other PC axes. In this sense, reduction to 50 PCs means selection of the first (most ‘important’) 50 PCs to approximate the complete PCA transformation, while omitting the (less important) rest of 466 PCs.

Figure R1. Schematic illustrates a complete PCA transformation. Selection of the first 50 PCs represents an approximation of the complete PCA transformation. The blue numbers in the parentheses indicate the real dimensions for LUAD cancer.

In PCA, the information carried by each PC decays dramatically over the index, i.e., the ‘importance’ of each PC diminishes one after another. The first few PCs typically explain much greater proportion of variance compared to those indexing after them (Figure R2). Particularly, the first three PCs can explain up to 90% of the total variance in some applications. That means working on the first few PCs can well capture the essential information contained in the original matrix, and at the same time reduce the time and computation cost significantly. Therefore, the choice of the number of PCs for dimension reduction is a balance of computational complexity and information retention rate. In our practice on the TCGA isoform expression data, the first 50 PCs explain 60-95% of total variance across cancer types, and we do see significant associations up to PC_50 (Figure S2A). Inspired by this comment, we further checked potential iSoMAs genes identified with PCs behind PC_50 (i.e., PC_51 – PC_100). Still in LUAD, with this setting our pipeline detected 241 additional ‘iSoMAs’ genes. However, even the most significant gene (TDRD6, Pmin=7.6e-6 at PC_89) of those 241 genes turned out to be in-significant upon validation with isoform expression levels (Figure R3). These analyses indicate that choice of 50 PCs represents a satisfactory balance between computational efficiency and information retention.

Figure R2. Proportion of variance explained by each individual PC in LUAD.

Based on above explanations, two scenarios exist with different selection of number of PCs:

First, if more than 50 PCs were selected, such as 100, the determination of both the iSoMAs gene itself and its target isoforms won’t change. This is because checking on more PCs potentially adds more significant PCs for the same gene but does not affect the original PCs in any way (neither the PC loading nor the PC score matrix). And because the importance of PCs decays over the index (see Figure S2F-I and Figure R3), we select the top-ranking PC (the one with smallest index) for downstream analysis when multiple significant PCs are identified by iSoMAs.

Second, if less than 50 PCs were selected, such as 10, this specific iSoMAs gene will not be determined any more if it were originally identified along PC axes after PC_10 (i.e., PC_11 – PC_50). In this case, this iSoMAs gene will be lost for the cancer under study, together with its predicted targets.

Figure R3. An example shows iSoMAs genes detected at PC_51 – PC100 has diminished biological relevance.

Taken together, the target isoforms and their PC loading weights will remain unchanged for the same iSoMAs gene with different settings of the PC number, provided this gene is eventually determined as an iSoMAs gene. However, this iSoMAs gene might be lost for this specific cancer type if checking on too few PCs. This is why a relatively large number of PCs (than used in general applications) is critical to retain the majority part of information contained in the original isoform expression matrix, which guarantees that all (at least most) iSoMAs genes can be identified by the pipeline.

c. The current iSoMAs analysis involves a nested, two-layer statistical test, like QTL analysis. In certain cancer types, the number of tests for the first layer, the mutant genes layer, can reach up to 19,162 (UCEC). For QTL calculations, a popular approach is to apply the Benjamini-Hochberg or Storey q-value method for the first layer of FDR control at the gene level, followed by permutation-based multiple corrections at the SNP level. The authors addressed the issue of multiple testing corrections. However, while strict correction was applied at the PC level using the Bonferroni method, the gene-level multiple testing correction seems absent.

Reply:

We acknowledge that this is an important point. And we clarify that the gene-level multiple testing correction was not neglected but investigated in a subtle way and shown in the original Table S2C (now Table S2B, see a screenshot below). Briefly, in each cancer type, we first obtained a minimum p-value along the 50 PCs (Pmin) for each test gene. Next, we conducted a traditional multiple testing correction on the list of Pmin’s of all test genes with the Benjamini & Hochberg (i.e., FDR) method. After that, we checked the genes satisfying FDR<0.05 (FDR05). We found that the FDR05 genes completely contain our iSoMAs genes detected as Pmin<0.001 (or Bonferroni corrected adj.P<0.05), and the number of FDR05 genes turned out to be much larger than the number of our iSoMAs genes across all 33 TCGA cancer types. Even at the significance level of FDR<0.01 (FDR01), we obtained many cancer types that showed obviously more candidate genes than using our original iSoMAs scheme. This implies that our strict correction on PCs instead of on mutant genes guaranteed a low false positive rate.

To further address this concern, we performed an additional (secondary) multiple testing correction at the mutant gene level, i.e., a further correction on the obtained iSoMAs genes in each cancer type. Interestingly, all iSoMAs genes passed this additional correction with FDR<0.05 (see added Table S2F). This means that our iSoMAs genes passed a strict two-layer statistical test as the reviewer suggested. To better reflect this consideration, we modified the schematic workflow of the iSoMAs pipeline by adding the two steps of the p-value correction (Figure R5).

Figure R4. Screenshot of Table S2B shows a detailed summary of the iSoMAs analysis results for each cancer type and comparison among different multiple testing correction methods.

Figure R5. A modification of the iSoMAs pipeline (Figure 2A) by adding the two steps of p-value correction.

2. The iSoMAs process is efficient but lacks interpretability.

a. PCA is a linear dimensionality reduction method. It does not generate meta-isoforms based on prior knowledge like gene/isoform interactions but just maximizes variance retention among samples and produces orthogonal PCs. As a result, biologically implausible isoform combinations may arise, and this issue has not been thoroughly discussed. What is the actual biological explanation of cross-chromosome meta-isoforms? Why are the first 100 isoforms considered targets of mutant genes? Do these isoforms interact with each other? Can PCA capture isoforms of the gene itself or its interacting genes into a single PC and assign them large weights?

Reply:

Thanks for these insightful comments regarding the application of dimension reduction method (PCA) in a real biological question.

While PCA does not generate meta-isoforms based on prior knowledge like gene/isoform interactions, we aim to discover the biological foundations (i.e., that ‘prior knowledge’) underlying this mathematical representation through our iSoMAs analysis. We also believe there is a biological reason why those top (say, 100) isoforms aggregated together under the PCA decomposition. Strikingly, we do obtain some important insights related to those cross-chromosome isoforms composing each meta-isoform through a series of downstream bioinformatics analyses. In particular, the top isoform targets of some iSoMAs genes, although located in distance (many even cross-chromosome), can participate in the same or similar signaling pathways (Figure 4B, Figure 8D), representing interaction with each other. And iSoMAs identifies such kind of functional networks in an innovative way.

In another scenario, the top isoforms do not work in the same pathway and do not interact with each other, they are just co-targeted by the same mutant gene. This is also reasonable considering that our identified iSoMAs genes are enriched for splicing/transcription regulators such as RNA-binding proteins (RBPs), splicing factors (SFs) and transcriptional factors (TFs) (Figure 5). These splicing/transcriptional factors typically work at the genome scale and hence affect the processing and production of any transcript from genome wide.

Based on these explanations, we further conclude that in both scenarios, an iSoMAs gene can be associated with the isoform expression of itself. And in the first scenario, PCA can capture the isoforms of the gene itself and its interacting genes into a single PC with large weights to form a functional network. To look at a specific example, FGFR3 mutation proved to be associated with the expression of its own isoforms on different directions and at different significance levels, in both BLCA and KIRP cancers (Figure R6). We have added Table S3C to list all predicted self-targeted iSoMAs genes in each cancer type.

We further clarify that the specific number of top isoforms chosen for downstream analysis is not biologically essential. Since the isoforms were ranked by the PC loadings, which quantitatively reflects the contribution of each isoform to the overall difference in the PC scores (the ‘expression levels’ of the meta-isoforms). Therefore, as the PC loading values get smaller, the associations of the isoforms on the list to the mutant gene get weaker and at some point, will be negligible. There might be more or less than 100 isoforms really associated with the same mutant gene, which subjects to validation as shown in Figure 2I, Figure S2D-E, Figure 7, and Figure 8C. We tried to start with a number suitable for a meaningful downstream analysis, such as enrichment profiling. Also, the PC loadings typically decay in an exponential-like pattern over the index and show an obvious turning point around 100 (Figure 2G).

Figure R6. Example of self-targeted iSoMAs genes in representative cancer types.

b. When using PCA for dimensionality reduction, we do not treat each PC equivalently because they explain varying proportions of the total variance in the data. The first few PCs may explain up to 90% of the variance. However, iSoMAs does not differentiate between PCs. Any gene significant in one single PC will be considered an iSoMAs gene. Compared with iSoMAs genes identified with PC1, do iSoMAs genes identified with PC 40, PC 50, etc., truly exist and show a strong association with a specific isoform? We would like to see further analysis.

Reply:

Thanks for this constructive comment. We completely agree that each PC shouldn’t be treated equivalently when using PCA, since the first PCs explain a much greater proportion of variance than those ranking after them. In our iSoMAs pipeline, we take this into account by selecting the top-ranking PC (the one with smallest index) for prioritizing isoform targets when multiple significant PCs are obtained, which means that we do differentiate between PCs. One specific example is TP53 in LUAD, where TP53 was tested significant along four PC axes including PC_2, PC_4, PC_5 and PC_50, but we chose PC_2 axis to prioritize its associated isoforms (Figure 2F-I).

Our analysis shows that iSoMAs genes could exist along any of the 50 PC-axes, not just PC1 or the first few PCs, as evidenced by the nontrivial percentages represented by all the 50 PCs (Figure 2D-F; Figure S2A). But as we explained above, the distinguishing power decreases over the PC index, i.e., the iSoMAs genes identified with a large index of PC might be less strongly associated with the predicted targets. To specifically address this comment, we further checked the real association between the iSoMAs genes identified with PC_40 and PC_50 and their predicted isoform targets in a representative cancer. Checking the iSoMAs genes detected in LUAD, we found that seven iSoMAs genes (GABRQ, OR5M8, PRAMEF19, SYN3, MYO1F, RNF43 and RBL2) were identified with PC_40 and five iSoMAs genes (CDH8, TBX20, PDGFD, PRR30 and DEFB110) were identified with PC_50. Our additional analysis confirmed that all of them showed significant association with the expression level of many predicted targets. Figure R7 illustrates the detail of mutation-isoform expression association for representative genes picked from the abovementioned lists. These examples consistently verified that iSoMAs genes identified with a large PC index such as PC_40 and PC_50 truly exist and show a strong association with their predicted target isoforms, although the number of significantly associated isoforms and the general significance levels are decreased compared to their PC_1/PC_2 counterparts (Figure S2D).

Figure R7. Representative iSoMAs genes identified with PC_40 (A) and PC_50 (B) and the association with their predicted target isoforms in LUAD cancer. Comparison was conducted between wildtype and mutant group of each iSoMAs gene as indicated. ****P<1e-4, ***P<1e-3, **P<0.01, *P<0.05 with two-sided Wilcoxon rank-sum test.

c. The lack of interpretability is also reflected in the absence of a preference for cis/trans regulation of iSoMAs genes. In traditional QTL analysis, cis QTLs are usually significantly stronger than trans QTLs and are easier to identify. This is not only because cis effects are methodologically easier to detect, but also biologically more plausible. The physical proximity of a variant to a specific gene/isoform is naturally more likely to affect the expression of that gene/isoform. While iSoMAs can capture both cis and trans effects, it is surprising that iSoMAs do not capture relatively more cis effects, which should be more significant and easier to detect biologically. We hope to see more analysis comparing the iSoMAs with the more classic approach: directly associating SNV grouping of a specific gene with the expression of the target isoforms.

Reply:

Thank you for sharing these in-depth thoughts regarding the biological interpretation of mutation-isoform expression association. While similar to traditional eQTL analysis in terms of purpose, iSoMAs pipeline is different from eQTL analysis in both purpose and methodology. As we clarified in the Discussion, SNVs on iSoMAs genes might be better interpreted as isoform-level expression quantitative trait loci, or isoQTLs.

Considering that eQTL is a distance-based measurement on a chromosome, trans-eQTLs are harder to determine because the effect may be diluted over distance. On the contrary, iSoMAs captures the expression/splicing events impacted by a mutant gene, which can be locally but more likely, distantly located in a genome-wide manner. As explained above, our identified iSoMAs genes are enriched for splicing/transcription regulators such as RNA-binding proteins (RBPs), splicing factors (SFs) and transcriptional factors (TFs) (Figure 5). These splicing/transcriptional factors typically work at the genome scale and can affect any transcript across the whole genome. In many cases, a mutant gene (iSoMAs gene) can even impact downstream targets in an indirect way. A striking example of indirect regulation is observed in the effects of mutant TP53 on RNA splicing, which is achieved through the upregulation of an intermediate RBP called hnRNPK (ref. 18). Therefore, the mutation of a splicing/transcription factor is much more likely to impact the expression of isoforms of other genes compared to itself, leading to the observation that iSoMAs genes occur in trans more often than in cis.

While it’s technically feasible to exhaust all combinations of mutant gene and isoform expressions with the classic approach, the advantage of iSoMAs lies in its high efficiency in computation and novelty for false positive control. We have verified with classic approach (directly calculating the association) that the top 100 isoforms are significantly associated with their corresponding iSoMAs gene TP53 in LUAD cancer (Figure S2D and added Figure 2I, see also Figure R8 below). To completely address this comment, we further calculated the associations between TP53 mutation and all 3,315 input isoforms in LUAD cancer. The results consistently showed that the top-ranked isoforms are far more likely to be significantly associated with TP53 mutation compared to lower-ranking isoforms. We have added Table S2C to list these details.

In short, we interpret the group of isoforms associated with a mutant gene to represent a comprehensive network of regulatory and functional dependencies that incorporate the complexities of gene regulation.

Figure R8. Added Figure 2I shows that the top-ranked isoforms are more likely to be significantly associated with the corresponding iSoMAs gene TP53 compared to lower-ranking isoforms in LUAD cancer.

Minor comments
1. Some of the figure labels are incorrect, such as in Figure 2a:
- Multi-isoform: n! 59,866
- Original: n! 73,599
These annotations should be corrected.

Reply:

We have corrected these visualization errors.

2. Wording
In the wet lab validation part, it is well established that TP53 is a cancer-related gene. More discussion directly related to iSoMAs may be needed and some of the current wording and expressions are a bit unclear.
For example, why do we need to check the expression of TPX2, and NCAPG isoforms? Are these isoforms the top target isoforms of TP53? How do they rank among the top 100 targets? It’s further discussed in the supplement figure S8A but not clearly explained in the main text.

Reply:

We appreciate the opportunity to clarify the text. TP53 turned out to be the most prevalent iSoMAs gene across cancer types, since it was detected as an iSoMAs gene in 18 out of the 33 TCGA cancer types studied (Figure 2C).

With our RNA-seq of lung cancer cell line, we checked all 73,599 transcripts (same annotation used by TCGA) rather than TPX2 and NCAPG alone. Isoforms especially the top ones showed great consistency between our RNA-seq and TCGA data (Figure S2D and Figure S8A). We picked two representative targets, i.e., TPX2 and NCAPG, to visualize the details of differential expression profile. Indeed, these two genes represent the top targets of TP53 in LUAD cancer based on feature (PC) loadings, which ranked No. 1 and No. 4 respectively (Figure 2H). We have added these additional explanations in the revised manuscript.

Reviewer #2: The authors proposed a method to detect mutation-isoform expression association. It starts by performing PCA on the isoform expression matrix, and then conducts a differential PC score analysis by investigating the association between gene mutation status and the PC score of isoform expression. For each mutant gene, the top ranked isoforms (those with large PC loadings of the most significantly associated PC score) is selected for downstream analysis. Overall, the methodological contribution is limited, parameter settings of the method seem to be arbitrary, and the advantage of the method is not compared with state-of-the art methods. My concerns are provided below.

1, The method aims to detect mutation-isoform expression association by performing differential PC score (i.e. a weighted linear combination of original isoforms) analysis of isoform expression between mutant genes and the wild type.

(1) For each gene, the authors consider only the most significantly associated PC score, which obviously leads to information loss since the remaining PCs may also be associated with the gene. How the isoforms corresponding to the other PCs are associated with the gene are not assessed.

(2) For each gene, the top ranked 100 isoforms associated with large PC loadings are deemed as target isoforms. The significance of the association between individual isoforms and the gene is not statistically evaluated.

(3) In PCA, the direction of the PC score only represents the direction with maximal variance; therefore, the direction of PC score does not necessary most correlate with the gene status (mutant or wild type); this might compromise the statistical power of the method to detect mutant-isoform expression association.

Reply:

Thanks for these relevant and constructive comments.

(1) *“For each gene, the authors consider only the most significantly associated PC score, which obviously leads to information loss since the remaining PCs may also be associated with the gene. How the isoforms corresponding to the other PCs are associated with the gene are not assessed.”*

We first clarify that when multiple PCs test significant for an iSoMAs gene, we chose the first significant PC (i.e., the one with smallest index) for prioritizing target isoforms. This holds throughout all downstream analyses unless otherwise stated (e.g., Figure 2E). We have rewritten the relevant paragraph to accurately declare this point.

We further clarify that in most cases, the first significant PC turned out to be the most significant PC. This makes sense because in PCA, the information carried by each PC decays over the index (see Figure R9A below). In other words, the importance of each PC diminishes over the index. One specific example involving multiple significant PCs is TP53 in LUAD, where TP53 was tested significant along four PC axes including PC_2, PC_4, PC_5 and PC_50, but we chose PC_2 axis to prioritize its associated isoforms (Figure 2F-I, see also Figure R9B below).

Figure R9. (A) Information carried by each PC decays over the index. (B) When multiple significant PCs for an iSoMAs genes are obtained, the first one was chosen to prioritize its target isoforms.

At the PCA step of our iSoMAs pipeline, the input isoforms are prioritized (or ranked) for 50 times, each corresponding to one PC axis. That’s to say, “the isoforms corresponding to other PCs” are exactly “the same input isoforms corresponding to the chosen PC”. They are just prioritized in different ways, each along one of the 50 PC axes. For TP53 in LUAD, the top isoforms ranked by PC loadings along PC_2 (the chosen PC) showed much more significant association with TP53 mutation compared to the top isoforms prioritized by other significant PCs (PC_4, PC_5 and PC_50). Therefore, our iSoMAs pipeline can identify those really significantly associated isoforms.

Figure R10. The first significant PC of an iSoMAs gene prioritizes its target isoforms more effectively compared to other significant PCs. Heatmaps show the scaled expression levels of the top 50 positive (rows 1-50) and top 50 negative (rows 51-100) isoform targets of TP53 for all LUAD tumor samples ordered by their PC scores along different PC axes as indicated. The isoforms were ranked by the PC loading values along corresponding PC axis as indicated. The red and green triangles represent the same isoforms in the correlation and association plots for each PC axis. See Figure 2H-I and Figure S2H-I for details.

(2) *“For each gene, the top ranked 100 isoforms associated with large PC loadings are deemed as target isoforms. The significance of the association between individual isoforms and the gene is not statistically evaluated.”*

Indeed, we don’t evaluate the mutation-isoform expression association directly as the traditional methods does. Instead, we prioritize the isoforms associated with a given iSoMAs gene indirectly based on the PC loadings relevant to the significant PC axis, which is the novelty and major contribution to the field of our iSoMAs pipeline. This innovative approach is mathematically reasonable owing to the linear combination property of the PCA decomposition. We have demonstrated the great consistency between our iSoMAs pipeline and the classic direct association analysis with many examples, see Figure S2D-E, added Figure 2I, Table S2C and Figure R10 above for details. These analyses represent the direct statistical evaluation of the significance of the association between individual isoforms and the associated mutant gene. Most importantly, a differential PC score analysis is much more efficient than the direct differential isoform expression analysis in identifying the significant associations between gene mutation and isoform expression.

(3) *“In PCA, the direction of the PC score only represents the direction with maximal variance; therefore, the direction of PC score does not necessary most correlate with the gene status (mutant or wild type); this might compromise the statistical power of the method to detect mutant-isoform expression association.”*

It is true that the direction (sign) of PC score does not correlate with the gene status. We realized this and we did not have any assumptions on the (biological) correlation between the sign of PC score and the mutation status of the iSoMAs gene. Instead, we attempted to test the potential synergy effect in biology between two (mathematically) associated variables – the gene mutation and its corresponding PC score (Figure 4E; Figure S4B, S4D). The synergy hypothesis regards how the gene mutation and PC score co-correlate with the clinical phenotype (i.e., patient survival), rather the correlation between the two variables themselves. Specifically, we demonstrated that the gene mutation and PC score of its target isoforms together represent the clinical phenotype better than the gene mutation or the PC score alone.

On the other hand, in iSoMAs pipeline the relationship between the gene mutation status and the sign of its corresponding PC score plays no role in determining the mutation-isoform expression association. This can be clearly seen from the TP53 in LUAD example (Figure 2F), where the PC scores take both positive and negative values in both TP53-wildtype and mutant groups. In addition, we prioritize target isoforms based on the absolute value of the PC loadings, regardless of their sign. A larger PC loading (either positive or negative) contributes more to the overall PC score difference.

2, A simple and necessary baseline is to perform differential isoform expression between mutant and wild type genes. Benchmarking against this baseline and existing methods were not conducted.

Reply:

Thanks for raising this critical point. We clarify that throughout this work we performed a series of verifications with the classic approach by directly calculating the mutation-isoform expression associations (e.g., Figure S2D-E,L; Figure 8B-C, etc. See below Figure R11 for a screenshot of Figure S2D). To further address this concern, we added Figure 2I and Figure S2I to show more examples of direct validation with gene mutation and isoform expression data. These data represent various types of benchmarking with the baseline.

Figure R11. A screenshot of Figure S2D shows that the predicted isoform targets of TP53 are verified to be significantly associated with TP53 mutation status. Significance levels were derived from Wilcoxon rank-sum test, *****P*<1e-4, ****P*<1e-3, ***P*<0.01, **P*<0.05, ns: not significant.

As we explained in the manuscript, currently the literature lacks a comprehensive mutation vs. isoform expression association study (especially in pan-cancer) like iSoMAs, which prevents a systematic comparison between our approach and others. However, there indeed exist piecemeal studies of mutation-splicing (isoform expression) associations, which appeared to well support our iSoMAs results, especially on several well-known splicing factors such as SF3B1 and RBM10 (Figure 7; Figure S7). We also designed a proof-of-principle study in our wet-lab with lung cancer cell lines and RNA-seq to validate our pipeline predictions on TP53, the most prevalent iSoMAs gene in pan-cancer. The experimental data further supported our computational findings (Figure 8; Figure S8).

3, The authors identified more than 2,000 iSoMAs genes in STAD and UCEC cancers which are known for hypermutability, but none in CHOL and KICH cancers. Can significant associations be detected in CHOL and KICH cancers using conventional differential isoform expression (not using PCA) or other methods?

Reply:

Thanks, this is a good question to provide some insights into our findings. There may be weak associations that do not meet the statistical thresholds we set. We clarify that this does not mean that there inherently isn’t any iSoMAs genes for human CHOL and KICH cancers. We can only declare that no statistically significant associations were obtained based on the TCGA data available for these two cancers.

Specifically, as shown in Figure R12A (added Table S2E), there are only 10 and 3 genes that were eligible (i.e., mutant in at least 2 different samples) for the iSoMAs analysis for CHOL and KICH cancers, respectively. In addition, the number of mutant samples for each eligible gene are too small to yield a significant association (all the Pmin’s failed to exceed the threshold of 0.001). Technically, we can further our iSoMAs analysis for CHOL and KICH cancers with a looser threshold for Pmin instead of the current 0.001. But we couldn’t expect a strong statistical significance/reliability based on the available data. We have added explanations regarding the CHOL and KICH cancers in the revised manuscript.

Therefore, the too small number of mutant samples in CHOL and KICH cancers for every gene makes it extremely hard to detect significant associations with any mathematical methods. Indeed, our further investigation with the conventional differential isoform expression analysis method showed that very few (<5 out of the 59,866 candidate) isoforms displayed significant association with any of those eligible genes in CHOL cancer and none in KICH cancer (Figure R12B).

Figure R12. (A) A screenshot of Table S2E shows the mutant genes eligible for iSoMAs analysis for CHOL and KICH cancers. (B) Results of direct mutation-isoform expression association analysis with conventional approach on the mutant genes listed in (A). The p-values were derived from two-sided Wilcoxon rank-sum test and adjusted with the FDR method.

4, Arbitrary parameter settings. For example, why only one (i.e. the most significantly associated) PC score is considered? Why is the number of selected isoforms for the considered PC is set to 100? These settings do not have experimental support.

Reply:

We appreciate these useful comments and have addressed them by justifying the parameter selection wherever possible.

For the specific examples mentioned:

(1) We chose the first significant PC (which typically is the most significant PC for multi-PC cases, see detailed explanations for answering comment 1) because in PCA the information each PC carries decays over the index. With the specific example (TP53 in LUAD) we demonstrate that it is unlikely to detect any significant associations at large indices of PC axes (Figure R10).

(2) We clarify that the specific number of top isoforms selected for downstream analysis is not biologically essential. We ranked all input isoforms by the PC loadings obtained through the PCA transformation. This ranking directly reflects the contribution/importance of each isoform to the overall PC score difference. That’s to say, as the PC loading values get smaller, the associations of the isoforms on the list to the mutant gene get weaker and at some point, will be negligible. Therefore, only the top-ranked isoforms deserve further investigation. There might be more or less than 100 isoforms really associated with the same mutant gene, which warrants validation as done in Figure 2I and Figure S2D-E. We tried to start with a number suitable for a meaningful downstream analysis like enrichment profiling, as well as maintain consistency across cancer types.

Reviewer #3: The article “iSoMAs: Finding isoform expression and somatic mutation associations in human cancers” by Tan et al describes the development of a novel computational pipeline called iSoMAs (iSoform expression and somatic Mutation Association). This pipeline integrates DNA-seq and RNA-seq data to identify associations between somatic SNVs and isoform expression profiles in different cancer types. Using PCA, iSoMAs assesses the association between SNVs and the overall transcriptome expression pattern, rather than direct one-to-one associations. Applied to 33 cancer types from the TCGA database, iSoMAs identified 908 genes whose mutations are linked to altered isoform production in multiple cancer types, as well as additional genes specific to one or two cancer types. These genes include known oncogenes, tumor suppressors, and various regulatory proteins, highlighting their biological and clinical importance.

Authors validated the findings through independent studies, including the reversal of TP53 mutation effects in lung cancer cells. iSoMAs is noted for its computational efficiency and comprehensive modeling of cancer cell systems biology, making it the first PCA-based method to explore the association between somatic SNVs and transcriptome-wide isoform expression across various cancers.

The article is well-written, presenting its methodologies and findings with clarity and precision. The analyses are very comprehensive, offering a thorough exploration of the associations between somatic mutations and isoform expression profiles across multiple cancer types. I only have minor comments/suggestions for improvement:

Explain acronyms such FPKM, RSEM as soon as they appear.

Figure 8H and 8I – text is too small to read

Reply:

We appreciate your valuable comments on our work and manuscript. They are very constructive and encouraging to us.

We have carefully proofread our manuscript and explained all acronyms such as FPKM and RSEM when they first appear.

We have manually added larger texts to 8H and created a figure legend to better illustrate the phases. For 8I, since we have quantitatively summarized the numbers with point-line plots and a clear figure legend, the flow images are only for illustration purpose. We also added a comprehensive label for all plots for 8H and 8I.

Given the potential of iSoMA to indicate e.g., chromatic accessibility, while pairwise gene interactions provide valuable information, multiple gene interaction analysis offers a deeper and more holistic view of the regulatory mechanisms within biological systems. Perhaps, adding a discussion paragraph on how these could be done and help identify and understand the mechanisms underlying disrupted chromatin accessibility would be helpful.

Reply:

This is an important topic which deserves a deep analysis of its own. From our computational analysis of the sample-matched RNA-seq/DNA-seq/ATAC-seq data, we observed alterations of chromatin accessibility at both the iSoMAs gene and its predicted targets loci. Mutations in splicing factors such as SF3B1, U2AF1, and SRSF2, commonly found in certain cancers, have been reported to lead to widespread changes in splicing patterns and subsequent chromatin accessibility alterations. There could also be feedback loops wherein disrupted splicing can alter Pol II elongation rates, which in turn affects the recruitment of chromatin remodelers and histone modifiers. This can lead to changes in nucleosome positioning and chromatin accessibility. Indeed, this complicated regulatory network might be further investigated from the perspective of co-profile of chromatin accessibility with the ATAC-seq data. As suggested, we have added one paragraph to discuss the potential for further analysis of co-profiles of chromatin accessibility of multi-gene sets instead of only mutant-target gene pairs:

*One potential direction for further investigation can be extending our current multiomics (DNA-seq/RNA-seq/ATAC-seq)-based co-profile analysis from mutant-target gene pairs to multi-gene sets. One multi-gene set can be composed of different iSoMAs genes targeting the same isoform, different isoforms targeted by the same iSoMAs gene, or iSoMAs genes/iSoMAs targets that participate the same signaling pathway, etc. This extension could be straightforward because the association analysis for each constituent gene of the gene set is same with our current analysis for each member of the gene pair. The co-profile of chromatin accessibility of these functionally associated gene sets (instead of gene pairs) offers a deeper and more holistic view of the regulatory mechanisms within biological systems. It would elucidate the detailed interplay among gene somatic mutation, chromatin accessibility and isoform diversity/expression in the context of human cancer.*
