# Supplementary material for: iSoMAs: Finding isoform expression and somatic mutation associations in human cancers
Source: PLoS Comput Biol. 2025 Mar 7;21(3):e1012847. doi: 10.1371/journal.pcbi.1012847 (PMC12052144; doi:10.1371/journal.pcbi.1012847)
Supplement: Attachment — Submitted filename: Response_Letter_auresp_2.docx [file pcbi.1012847.s019.docx]

**Response to reviewers’ comments**

Reviewer #1: This revision represents a significant improvement upon the original submission, and we appreciate the effort the authors have invested in thoroughly examining the behavior of their method. Below are some specific comments and questions:

1.a
This is sufficiently addressed.

1.b
The authors mentioned that TDRD6, the most significant potential iSoMAs gene identified with PCs behind PC_50 in LUAD, turned out to be in-significant upon validation with isoform expression levels.

We have two inquiries regarding this:
1. In the lower-left corner of Figure R3, two isoforms show significant differences. Why is it stated that there is no significance at the isoform level? Is this due to the majority of isoforms being non-significant?

Reply:

We apologize for the confusion in our previous statement that, "TDRD6 turned out to be insignificant upon validation". What we meant to clarify is that while TDRD6 tested significant at a PC above PC_50 (Pmin=7.6 x 10^−6^ at PC_89), this example demonstrates that higher-index PCs (e.g., PC_89) provide weaker support in terms of the number of validated significant isoforms compared to lower-index PCs (e.g., PC_2 for TP53) (Figure R1A,B). Specifically, in LUAD, the majority of the top 100 predicted isoforms of TDRD6 (98%) show non-significant associations with TDRD6 mutation. In contrast, for TP53 in LUAD, only 2% of the top 100 predicted isoforms lack significant associations with TP53 mutation (Figure R1A).

Figure R1. An example shows iSoMAs genes detected at PC_51 – PC100 have fewer downstream targets compared to those detected at PC_1 – PC_50 (TDRD6 vs. TP53 in LUAD). For both differential PC and isoform expression, Wilcoxon rank-sum test, *P<0.05, **P<0.01, ***P<0.001, ****P<1e-4.

2. For the iSoMAs genes identified within the PC_01 to PC_50 range, has isoform-level significance also been validated using the same method, especially for the candidate genes identified in the later PCs (such as PC_41 to PC_50)?

Reply:

Yes. We performed a series of validations for other iSoMAs genes identified with the PC_1 to PC_50 range with the same method, i.e., the classic statistical analysis by two-sided Wilcoxon’s rank-sum test between wildtype and mutant groups, using the TCGA somatic mutation and isoform expression data directly. Examples include the association between TP53 mutation and expression of all 3,315 input isoforms in LUAD cancer (Table S2C, Figure S2D-E, H-I), the associations between mutation of some splicing factors (SF3B1, RBM10) and isoform expression of their predicted target genes (Figure 7 and S7), etc. Since we have mathematically proven that our pipeline with dimension reduction can accurately identify those mutation-isoform expression associations in an efficient way, the purpose of those example validations was to showcase that iSoMAs really works.

In PCA, the distinguishing power decreases over the PC index, i.e., the iSoMAs genes identified with larger PC indices might be less strongly associated with the predicted targets (i.e., the top isoforms ranked by PC loading). We also specifically verified the real association between the iSoMAs genes identified at the later PCs (e.g., PC_40 and PC_50) and their predicted isoform targets in a representative cancer. Briefly, in LUAD we found that seven iSoMAs genes (SYN3, OR5M8, PRAMEF19, RBL2, MYO1F, RNF43 and GABRQ) were identified with PC_40 and five iSoMAs genes (PRR30, CDH8, PDGFD, DEFB110 and TBX20) were identified with PC_50. This additional analysis confirmed that all of them showed significant associations with the expression level of many predicted targets (Figure R2, see details in added Table S2G). However, it deserves noting that the number of significantly associated isoforms and the general significance levels can be decreased compared to their counterparts detected at PCs of smaller indices (see the TP53 case in Figure R1).

Figure R2. iSoMAs genes identified with PC_40 (A) and PC_50 (B) and the association with their predicted target isoforms in LUAD cancer. Comparison was conducted between wildtype and mutant group of each iSoMAs gene as indicated. ****P<1e-4, ***P<1e-3, **P<0.01, *P<0.05 with two-sided Wilcoxon rank-sum test.

1.c
The authors stated the gene-level multiple testing correction was not neglected but investigated in a subtle way. We would appreciate further clarification on this.

1. The authors indicated that the first step of iSoMAs involves obtaining the minimum p-value across the 50 PCs (Pmin) for each test gene. If the first significant PC (i.e., the PC with the smallest index among all significant PCs) was selected for downstream analysis, should the p-value of this first significant PC (Pfirst), instead of the Pmin be used for multiple testing correction? Given that different PCs represent different meta-isoforms, we suppose these two cannot be conflated.

Reply:

We thank the reviewer for this opportunity to clarify the process. We need to emphasize that our study has two separate and independent steps. For the first step, which has the purpose of detecting the iSoMAs genes, we choose one p-value for each mutated gene, specifically the one with the minimum value, denoted as Pmin. We utilize the the Pmin to ensure we catch the breadth of possible iSoMAs genes. The Pmin must satisfy a predefined threshold after Bonferroni correction to account for testing 50 PCs for each gene (Figure 2A).

The second step involves testing the differential isoform expression between wild type and mutant iSoMAs genes. For this step we attempt to use a PC with a low index because lower indexed PCs capture larger fractions of variance (this decreases dramatically for higher indexed PCs) and therefore have potential for revealing the highest biological impact. For this reason, we use the lowest indexed PC that passes the significance threshold, i.e., the Pfirst. We note and emphasize here that for the majority of iSoMAs genes the PCs associated with Pfirst and Pmin are the same (updated Table S2B, see also Figure R3 below). Specifically, in 26 out of the 31 cancer types with iSoMAs genes detected, Pmin = Pfirst more than 90% of the time (92% across all cancer types). In the rare cases when the PCs associated with Pfirst and Pmin are different, we choose the PC associated with Pfirst for the differential isoform expression analysis in an attempt to capture a higher biological impact, as stated above.

Figure R3. Updated Table S2B. PanCan3: number of iSoMAs genes overlapping the 908 PanCan3 (present in at least 3 cancer types) iSoMAs genes in each cancer; N.Pmin: number of iSoMAs genes detected with Pfirst=Pmin; pct.Pmin: percentage of Pfirst=Pmin, calculated as N.Pmin/iSoMAs*100.

2. For the hierarchical two-layer correction procedure, the multiple hypothesis tests for candidate genes were controlled (Step 2, global correction) based on the multiple testing-adjusted statistics (Step 1, local correction) of each gene's best association, rather than the initial p-values. For iSoMAs, if the local correction is performed using the Bonferroni procedure, the second-level FDR should be applied to the adjusted p-values (Padj-Bonferroni) rather than the initial minimum p-values (Pmin). However, it appears that the current FDR05 set was generated with Pmin, which makes it a pooled single-layer correction procedure. We are concerned about this because the pooled FDR methods (even the strict Bonferroni procedure) often fail to control the FDR of target genes.

Reply:

Thanks for further discussion on this important point. As suggested, we have now applied the second-level FDR procedure to the Bonferroni-adjusted Pmin’s (Pmin.Bonf.FDR) and updated S2F accordingly. Our analysis results show that, although the two-layer corrected p-values Pmin.Bonf.FDR are typically larger than the single-layer p-values Pmin.FDR, they are still controlled by the level of FDR<0.05 (Table S2F, see Figure R4 for the table header). That means that the two-layer correction still retains the iSoMAs genes we originally identified. However, only a small portion of iSoMAs genes were retained after the secondary correction at the level of FDR<0.01. Since the iSoMAs genes were detected at different significance levels, they are not equally deserving for a further investigation. We kept all iSoMAs genes at the level of Pmin.Bonf.FDR<0.05 to offer the research community as many candidates as possible but recommend users to choose the top-ranked iSoMAs genes contingent on specific questions/applications. The major contribution of this work is to show that the mutation-isoform expression association can be detected in an indirect but much more computationally efficient way than traditional approaches. We have added these clarifications to the Discussion.

 Figure R4. Header of the updated Table S2F. We added the Bonferroni-corrected Pmin values (Pmin.Bonf), and their secondary correction results with FDR (Pmin.Bonf.FDR).

2.a
This is sufficiently addressed.

2.b
This is sufficiently addressed.

2.c
This is sufficiently addressed.

Reviewer #2: My previous comments were not well addressed, which are described below.

(1) Regarding point 2 of my first comment (“…The significance of the association between individual isoforms and the gene is not statistically evaluated”), the authors provide only an example using the TP53 gene (Figure S2D-E, added Figure 2I, Table S2C and Figure R10). A thorough benchmarking on the different types of cancers considered in this work is necessary (but not performed), because this is key to assess the advantage of IsoMA over conventional differential isoform expression analysis. Without such benchmarking, it is not sufficient to demonstrate of the power/contribution of ISoMA (which associations are uniquely identified by ISoMA? Which are unique to conventional methods? Which are shared by both?). Actually, taking Figure R12 in the response letter as an example, conventional methods do identify significantly differentially expressed isoforms that ISoMAs cannot detect.

Reply:

Following the reviewer’s suggestion, we have now performed a thorough validation and benchmarking on the different cancer types by conventional differential isoform expression analysis (Table S2G-J). Specifically, Table S2G lists all the significant associations among the top 100 isoforms for each iSoMAs gene in each cancer type that are validated by the conventional approach. Table S2H shows a benchmarking analysis to detect mutant genes associating with gene isoform expression and significant mutation-isoform expression associations with the conventional approach in comparison with the iSoMAs pipeline. Table S2I-J summarize the numbers of mutant genes associating with gene isoform expression and the numbers of mutation-isoform expression associations detected by iSoMAs and conventional (Conv) approach (see also Figure R5 below). A comparison of genes and associations determined by iSoMAs and Conv indicates that the methodological differences and multiple testing corrections are likely to give overlapping yet distinct results. There are iSoMAs genes recorded for more cancer types, whereas the conventional method typically has fewer mutant genes associating with fewer gene isoform expression differences, and the average/median numbers of associated isoforms are typically low, especially for the genes uniquely identified by the conventional method (Conv.uniq.mean, Conv.uniq.median). These results suggest that the iSoMAs pipeline catches more relevant genes and associations overall.

Figure R5. Screenshot of Table S2I-J shows the number of mutant genes associating with gene isoform expression (left) and the number of gene-isoform expression associations (right) detected by the iSoMAs pipeline and conventional approach (Conv, direct differential isoform expression analysis using Wilcoxon rank-sum test with FDR correction). iSoMAs.uniq: genes or associations uniquely identified by iSoMAs; Conv.uniq: genes or associations uniquely identified by conventional approach; Shared: genes or associations shared by both methods; Conv.mean (Conv.uniq.mean): average number of associations per mutant gene (uniquely) identified by conventional approach; Conv.median (Conv.uniq.median): median number of associations per mutant gene (uniquely) identified by conventional approach.

(2) Regarding point 3 of my first comment (“the direction of the PC score only represents the direction with maximal variance; therefore, the direction of PC score does not necessary most correlate with the gene status (mutant or wild type)”), here by “direction”, I mean the projection direction of the data that explain a maximal amount of variance (a detailed explanation of direction can be found elsewhere, such as <https://builtin.com/data-science/step-step-explanation-principal-component-analysis>), but not the positive/negative sign.

Reply:

Thanks for clarification of this point and offering the useful reference. We agree on the mathematical principles underlying PCA where the principal components represent the directions of the data that explain a maximal amount of variance, i.e., eigenvectors that capture most information of the data. We make full use of this principle to prioritize target isoforms in an indirect but efficient way. Specifically, we leverage the indexed PCs and the PCA decomposition to demonstrate that predicted target isoforms are altered in the presence of an iSoMAs gene mutation.

To explain further: A PC score is a linear combination of the original isoform expressions, effectively forming a "meta-isoform," where each isoform contributes a specific weight determined by the PC loading matrix. If a PC score – particularly among the first few PCs, which capture the most variance – is significantly associated with the gene’s mutation status, the isoforms contributing most to that PC also differ significantly between the wildtype and mutant groups.

Using the Wilcoxon rank-sum test, we can statistically test for significant differences in PC scores between the wildtype and mutant groups. Isoforms with large coefficients in the loading matrix are the primary contributors to the PC scores, making them more likely to be associated with the mutation status. This is exemplified in Figure 2H, where scaled target isoform expression differs distinctly between the mutant and wt TP53 genes. Similarly, Figure 2I shows that the top-ranked isoforms, as determined by their contributions to PC scores in TP53 for LUAD, exhibit the strongest associations with mutation status (Figure R6). These results demonstrate that differential PC analysis effectively identifies and prioritizes isoforms likely impacted by gene mutations.

Figure R6. A screenshot of Figure 2H-I.

(H) Scaled expression levels of the top 6 positive (rows 1-6) and top 6 negative (rows 7-12) isoform targets of TP53 for all LUAD tumor samples ordered by their PC scores along PC_2 axis. The isoforms were ranked by the PC loadings along PC_2 axis. (I) Left: Spearman correlation between PC_2 score and expression level of each of the input 3,315 isoforms across 517 LUAD tumor samples. Right: Association between TP53 mutation and expression level of each of the input 3,315 isoforms across 517 LUAD tumor samples, measured by two-sided Wilcoxon rank-sum test. The top 50 positive and top 50 negative isoforms (ranked by PC_2 loadings) are marked red and green, respectively.

Reviewer #3: The authors addressed my comments therefore I approve the revisions.
